# Crosstalk between NF-κB and Nucleoli in the Regulation of Cellular Homeostasis

**DOI:** 10.3390/cells7100157

**Published:** 2018-10-05

**Authors:** Jingyu Chen, Lesley A. Stark

**Affiliations:** Edinburgh Cancer Research Centre, Institute of Genetics and Molecular Medicine (IGMM) University of Edinburgh; Edinburgh EH42XU, Scotland, UK; Jc2037@cam.ed.ac

**Keywords:** Nucleolus, TIF-IA(RRN3), I-kappaB, stress, aspirin, CDK4, RelA, p65, cancer, neurodegenerative disorders, NF-κB, non-steroidal anti-inflammatory drugs (NSAIDs)

## Abstract

Nucleoli are emerging as key sensors of cellular stress and regulators of the downstream consequences on proliferation, metabolism, senescence, and apoptosis. NF-κB signalling is activated in response to a similar plethora of stresses, which leads to modulation of cell growth and death programs. While nucleolar and NF-κB pathways are distinct, it is increasingly apparent that they converge at multiple levels. Exposure of cells to certain insults causes a specific type of nucleolar stress that is characterised by degradation of the PolI complex component, TIF-IA, and increased nucleolar size. Recent studies have shown that this atypical nucleolar stress lies upstream of cytosolic IκB degradation and NF-κB nuclear translocation. Under these stress conditions, the RelA component of NF-κB accumulates within functionally altered nucleoli to trigger a nucleophosmin dependent, apoptotic pathway. In this review, we will discuss these points of crosstalk and their relevance to anti-tumour mechanism of aspirin and small molecule CDK4 inhibitors. We will also briefly the discuss how crosstalk between nucleoli and NF-κB signalling may be more broadly relevant to the regulation of cellular homeostasis and how it may be exploited for therapeutic purpose.

## 1. Introduction

NF-κB is the collective name for a family of inducible transcription factors that play a pivotal role in many cellular processes including immune response, inflammation, proliferation, and apoptosis [1,2]. In addition to classical stimuli such as cytokines and pathogens, NF-κB is induced by a plethora of environmental and cytotoxic insults [3]. The mechanism by which these multiple insults induce the pathway, and what determines the downstream consequences on proliferation and apoptosis, has remained unclear. However, recent studies suggest that nucleoli play a role. An atypical nucleolar stress response pathway has been identified that lies upstream of NF-κB signaling [4]. It has also been shown that following induction, the RelA component of NF-κB can accumulate in nucleoli to trigger apoptotic pathways [5,6]. This cross talk between NF-κB and nucleoli is important for the anti-tumour effects of aspirin and small molecule CDK4 inhibitors, suggesting therapeutic relevance. In this review, we will discuss nucleolar stress and the various levels of convergence between this and the NF-κB pathway. We will also discuss the relevance to the anti-tumor mechanisms of aspirin, CDK4 inhibitors, and other therapeutic agents. Finally, we will touch on other processes that may be regulated by this crosstalk.

## 2. The Nucleolus and Stress

**Figure 1:**
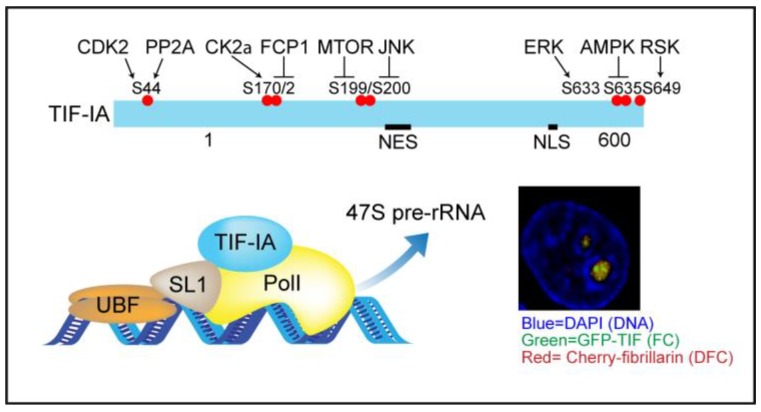
Top: TIF-IA is targeted by multiple kinase and phosphatase pathways. Bottom: the phosphorylation status controls the transcriptional activity of the pre-initiation complex, of which TIF-IA is a vital component. NES-nuclear export signal. NLS-nuclear localisation signal. Insert; confocal image showing the localisation of GFP-TIF-IA in fibrillar centres (FC) of nucleoli and cherry-fibrillarin in the surrounding dense fibrillar component (DFC) in fixed colorectal cancer cells. DAPI marks the DNA.

The nucleolus is a highly dynamic, membrane-less nuclear organelle [7]. It is primarily recognized for its role in ribosome biogenesis which is the most energy consuming process in the cell and as such, is tightly linked to metabolic and proliferative activity. The first stage is transcription of 47S pre-ribosomal RNA (rRNA) by RNA polymerase I (PolI) (Figure 1). This transcription requires the formation of a pre-initiation complex (PIC) which includes upstream binding factor (UBF) and SL-1: a multi-protein complex consisting of TBP and at least 4 PolI specific TAFs. SL-1 confers promoter specificity while UBF acts as a de-repressor and co-activator. Another critical component of the PIC is TIF-IA (gene name RRN3). This is essential for rDNA transcription as it forms a bridge between SL-1 and Pol I, thereby tethering PolI to the rDNA promoter to generate a functional transcription pre-initiation complex [8,9,10,11]. Maturation of 47S pre-rRNA occurs co-transcriptionally through a series of covalent modifications, nucleolytic processing steps and interactions with ribosomal, and accessory proteins. This generates pre-ribosomes, which are further matured to the 40S and 60S ribosomal subunits.

Nuceloi are divided into three sub-compartments namely; fibrillar centres (FC), dense fibrillar component (DFC), and granular component (GC). Transcription of rDNA takes place at the interface between FCs and DFCs while maturation takes place in the DFC and GC. Ribosomal DNA arrays are clustered in nucleolar organiser regions (NORs), which are present on the short arms of all five human acrocentric chromosomes [12]. NORs show various levels of activity and while nucleoli form around transcriptionally active NORs, inactive arrays are extra-nucleolar, embedded in and contributing to the heterochromatin that surrounds the organelle [13]. Generation and maintenance of the tri-partite nucleolar substructure is dependent on transcription of rDNA in active NORs and liquid-liquid phase separation (LLPS) of nucleolar components [13,14]. 

### 2.1. Nucleolar Maintenance of Cell Physiology

**Figure 2:**
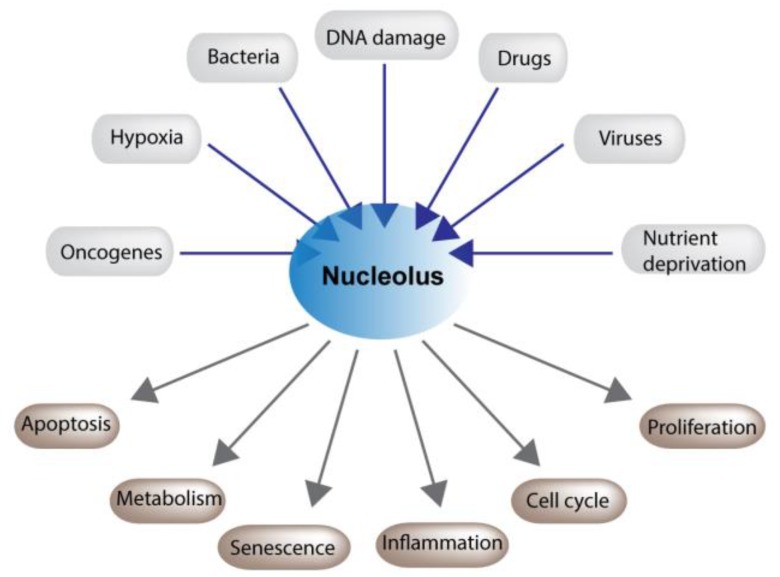
Nucleoli play a critical role in maintaining cellular homeostasis. Nucleolar function is altered in response to a plethora of cytotoxic and environmental stresses. This disruption modulates an array of cellular processes which allow cells to recover or, if the damage is to great, to undergo cell death.

In addition to its role in ribosome biogenesis, the nucleolus acts as a critical stress sensor and coordinates downstream responses to stress such as altered metabolism, differentiation, cell cycle arrest, autophagy, DNA repair, senescence, and apoptosis [15,16] (Figure 2). Perturbations in nucleolar function are associated with many common diseases including ischemic heart disease, neurodegenerative disorders and cancer. Nucleolar size and number have also recently been associated with longevity in c. elegans and mammalian models [17,18]. Indeed, it now appears that proper dynamic control of nucleolar activity is crucial for maintaining tissue homeostasis and health.

The rate limiting step in ribosome biogenesis is transcription of the 47S pre-rRNA, which is extremely sensitive to changes in cellular environment. If cells are exposed to harmful conditions, i.e., nutrient starvation, cytotoxic agents, physical insults, or viral infections, rDNA transcription is inhibited and there is a rapid and dramatic change in nucleolar structure: the FC and DFC become segregated from the GC and form “caps” at the nucleolar periphery [19,20,21]. A cascade of signaling events are also initiated that influence cell phenotype [7,22]. This process is broadly termed nucleolar stress and can take different forms, dependent on cell context and the nature of the insult [23,24]. Over 4500 proteins have been isolated from mammalian nucleoli and over half of these are involved in processes out with ribosome biogenesis [25,26,27]. It is thought to be the dynamic flux of these proteins between nucleoli and other cellular compartments in response to stress that is ultimately responsible for the downstream effects on cell physiology [25,26].

### 2.2. p53 Dependent and Independent Consequences of Nucleolar Stress

The signals that link nucleolar stress to changes in cell physiology are only beginning to emerge. The most characterized pathway is the MDM2-p53 axis, which is covered in depth in some excellent reviews [28,29,30,31]. Briefly, upon stress-mediated perturbation of ribosome biogenesis, ribosomal proteins (RP) L5 and 11 are released from nucleoli in a NEDD8/PICT1 dependent manner. These proteins then accumulate in the nucleoplasm and bind to the p53 E3 ligase, MDM2. This inhibits MDM2 activity, thus preventing the ubiquitination and proteasomal degradation of p53. Consequently, p53 is stabilized and activates target genes involved in cell cycle arrest, senescence, and apoptosis. While this pathway is clearly important, recent reports from yeast, flies and mammalian cells indicate that in some contexts, perturbation of ribosome biogenesis can modulate cell growth, death, and autophagy in a p53 independent manner [23,24,32,33]. For example, Donati et al. demonstrated that specific interference with the PolI factor, *POLR1A*, induces cell cycle arrest in mammalian cells in the absence of p53 [34]. They proposed a model in which RPL11 binding to MDM2 blocks the MDM2-E2F interaction, thus causing E2F degradation and cell cycle arrest. Similarly, the Russo lab demonstrated that the apoptotic response to nucleolar stress can occur in the absence of functional p53 [35]. In this study, RPL3 was overexpressed to mimic perturbations of ribosome biogenesis. This caused the formation of an RpL3, Sp1, NPM complex at the p21 promoter and consequently, cell cycle arrest and apoptosis. In another example, it was shown that nucleolar stress destabilizes the proto-oncogene PIM1, causing increased levels of p27Kip1 and cell cycle arrest in p53-/- cells [36]. Proteomic studies indicate that hundreds of proteins that shuttle from the nucleolus in response to cytotoxic stimuli have p53 independent functions, supporting the notion of important p53 independent nucleolar stress pathways [25,26].

## 3. TIF-IA-NF-κB Nucleolar Stress

Like p53, NF-κB plays a critical role in maintaining cellular homeostasis under stress and emerging evidence indicates this transcription factor pathway also lies downstream of perturbed nucleolar function [4].

### 3.1. Stress Activation of the NF-κB Pathway

In mammalian cells there are five members in the NF-κB family namely, RelA (p65), RelB, c-Rel, p105/p50 (NF-κB1), and p100/p52 (NF-κB2) [37,38]. These proteins homo and hetero-dimerize through their Rel homology domain to create a variety of transcription factor complexes (56). In resting cells, these complexes are retained in the cytoplasm by a family of IκB inhibitory proteins (IκBα, IκBβ, IκBγ and Bcl-3). When the cell is exposed to a wide array of stimuli including inflammatory cytokines, bacterial pathogens, cytotoxic agents, nutrient deprivation, hypoxia, and physical insult, IκB proteins are phosphorylated by inhibition of IκB (IKK) kinase (IKK1/IKKα, IKK2/IKKβ IKKγ/Nemo) complexes [37]. This phosphorylation targets IκB for ubiquitination and degradation by the 26S proteasome. NF-κB complexes are then free to translocate to the nucleus where they influence the expression of numerous (>150) genes including those involved in inflammation, immune response, senescence, cell cycle, and apoptosis [3].

Classic NF-κB stimuli such as tumor necrosis factor alpha (TNFα) and interleukin-1 (IL-1) induce rapid IKK activation/IκB degradation and the upstream pathway responsible for this rapid activation is very well documented [39,40]. In contrast, stress stimuli (including UV-C radiation, nutrient deprivation and chemopreventative/therapeutic agents) tend to activate the pathway with a much slower and delayed kinetic [3,41]. A number of mechanisms have been proposed for this delayed activation. For example, Kato et al. demonstrated that UV-C-mediated degradation of IκB is dependent upon a p38-CK2 axis [42], while Jiang et al. demonstrated that phosphorylation of translation initiation factor 2α (eIF2α) is required for activation of the NF-κB pathway by a variety of stresses [43,44]. More recently, it was shown that an atypical form of nucleolar stress, characterized by degradation of the PolI complex component, TIF-IA, lies upstream of NF-κB signaling in response to specific stress stimuli [4].

### 3.2. TIF-IA Degradation—A Novel Form of Nucleolar Stress

TIF-IA is the key component of the PIC that transduces environmental signals to the PolI transcriptional machinery [22,45]. If nutrient availability is altered or the cell is under stress, the phosphorylation status of TIF-IA is modulated by a complex network of kinases and phosphatases, which ultimately activate or inactivate the protein to fine tune the transcriptional output (Figure 1) [46]. While TIF-IA is mainly known for its role in the nucleolus, the protein shuttles dynamically between this and other cellular compartments. Indeed, using a GFP-TIF-IA approach, Szymański et al. found that 48% of the protein is present in the cytoplasm while only 7% is located in the nucleolus (although the concentration in the nucleolus is higher) [47]. The mechanisms that control the cellular localization of TIF-IA are still unclear, but are known to be targeted by specific stresses [48]. It is also unclear if the protein plays a role in other compartments. What is clear is that it is an important regulator of cell proliferation and apoptosis. Genetic deletion in mice leads to embryonic lethality while deletion or depletion in mouse embryonic fibroblasts (MEFs), cancer, and neuronal cells causes cell cycle arrest and apoptosis [49,50].

**Figure 3:**
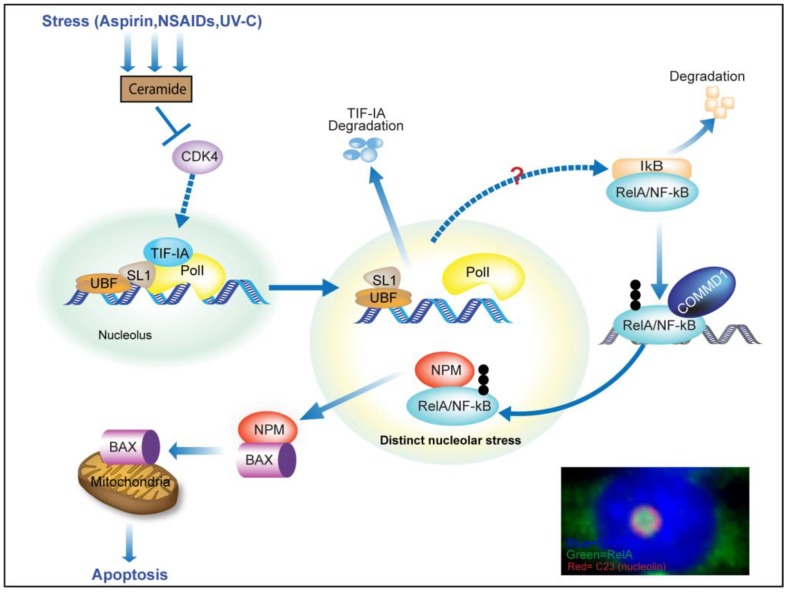
TIF-IA-NF-kB-nucleololar stress response. When cells are exposed to a variety of specific stresses, ceramide is generated leading to inhibition of CDK4. This inhibition induces degradation of TIF-IA (in a manner dependent upon UBF and p14ARF), which in turn causes increased nucleolar size, gross changes in nucleolar morphology and degradation of IkB. IkB degradation allows RelA/NF-kB to translocate into the nucleus and recruit a COMMD1 dependent ubiquitin ligase complex. Ubiquitination of RelA by this specific complex targets the protein to nucleoli, where it binds nucleophosmin (NPM), causing this protein to relocate out of nucleoli to the cytoplasm, where it is free to bind BAX and transport BAX to the mitochondria to mediate apoptosis. The signalling network(s) that links altered nucleolar function to IkB degradation is unknown. Inset: Immunomicrograph showing enlarged segregated nucleoli and nucleolar accumulation of RelA in response to aspirin (5mM, 16h). DAPI depicts DNA.

Given the considerable overlap in stresses that target TIF-IA/perturb ribosome biogenesis and those that activate NF-κB, our lab explored the connection. In doing so, we uncovered a novel pathway by which nucleolar function is altered by stress (Figure 3) [4]. We found that multiple stress stimuli, including aspirin, UV-C and the second messenger ceramide, not only alter the phosphorylation status of TIF-IA, but also induce degradation of the protein. This effect was not observed in response to TNF or the DNA damaging agent, camptothecin, indicating specificity. The mechanism by which TIF-IA is degraded in response to stress is complex and involves both proteasome and lysosomal pathways. It is dependent on de-phosphorylation of TIF-IA at Serine 44 and the PolI complex associated factors upstream binding factor (UBF) and p14ARF. It also lies downstream of CDK4 inhibition, which is a common response to stress stimuli of the NF-κB pathway.

As would be expected, stress-mediated degradation of TIF-IA was associated with inhibition of rDNA transcription. It was also associated with striking morphological changes to nucleolar structure and activation of the NF-κB pathway.

### 3.3. Nucleolar Enlargement as a Consequence of TIF-IA Degradation

In most cases, stress-mediated segregation of the nucleolar sub-structure is associated with a significant reduction in size of the organelle. A role for TIF-IA in this phenomenon was initially suggested by genetic deletion of the gene in MEFs, which caused a loss of nucleolar morphology and a reduction in nucleolar size [49]. Stress-mediated inhibition of TIF-IA by targeted phosphorylation is also associated with decreased nucleolar size [22,48]. In contrast, we found that stress-mediated degradation of TIF-IA is paralleled by a striking increase in nucleolar size, alongside segregation of nucleolar components [4] (Figure 3). This stress-mediated increase in nucleolar size was paralleled by inhibition of rDNA transcription and was blocked when TIF-IA degradation was blocked, indicating that the two events are linked. These data question the paradigm that nucleolar size is linked to the rate of rDNA transcription. Similar to these data, Fatyol et al. found that the MG132 proteasome inhibitor induces a significant increase in nucleolar volume while inhibiting rDNA transcription and inducing morphological changes to nucleoli [51]. Interestingly, we have found that low dose MG132 causes TIF-IA degradation in a similar manner to stress (unpublished data). The NEDD8 inhibitor MLN4924 has also been shown to cause an increase in nucleolar size alongside nucleolar stress [52].

### 3.4. Activation of the NF-κB Pathway as a Consequence of TIF-IA Degradation

The first evidence that NF-κB signaling may lie downstream of perturbation in nucleolar function came from experiments showing siRNA-mediated depletion of PolI complex components, (including TIF-IA) causes degradation of IκBα, S536 phosphorylation of RelA (a marker of activation), nuclear translocation of RelA, increased NF-κB transcriptional activity and increased transcription of NF-κB target genes [4]. Interestingly, this effect was not mimicked by the PolI inhibitors actinomycinD, CX5461 or BMH-21, suggesting that unlike p53 nucleolar stress response, activation of NF-κB signaling is not directly linked to inhibition of rDNA transcription. Kinetic studies revealed that stress-mediated degradation of TIF-IA precede cytoplasmic activation of NF-κB, suggesting a potential link. Indeed, it was found that blocking degradation of TIF-IA, using specific siRNAs and a dominant negative TIF-IA mutant, blocked the effects of specific stresses on the NF-κB pathway [4] (Figure 3). These data revealed a novel TIF-IA-NF-κB nucleolar stress axis.

The TIF-IA-NF-κB nucleolar stress response pathway was evident in multiple cell types and in tumors from colon cancer patients treated ex vivo with the chemopreventative agent aspirin (see below) indicating broad and *in vivo* relevance [4]. Multiple proteins that regulate the NF-κB pathway reside within nucleoli, which could account for this connection. Interestingly, CK2, which has previously been shown to be involved in UV-C-mediated activation of the NF-κB pathway [42], is bound to TIF-IA in the PolI complex [42,53]. Similarly, phosphorylation of eIF2α in response to ER stress has been shown to both inhibit TIF-IA activity [54] and to activate NF-κB [43,44]. NIK (NF-κB inducing kinase), which acts upstream of the IkappaB kinase (IKK) complex, shuttles through nucleoli [55]. The ribosomal proteins L3 and S3 have also been shown to complex with IκB and modulate NF-κB activity respectively [55,56,57]. L3 was found to bind to and stabilize IκB, thus repressing NF-κB activity, while S3 promoted activity by interacting with NF-κB complexes in the nucleus.

### 3.5. TIF-IA-NF-κB Nucleolar Stress and the Induction of Apoptosis

While stimulation of the NF-κB pathway is generally regarded as anti-apoptotic, in particular contexts, and especially in response to cellular stress, NF-κB acts to promote apoptosis [58,59]. Indeed, those stresses that stimulate the NF-κB pathway through TIF-IA degradation (eg aspirin, UV-C, ceramide) are known to require nuclear translocation of NF-κB for their pro-apoptotic activity [60,61,62,63,64]. In keeping with a pro-apoptotic role for the TIF-IA-NFκB pathway, it was found that blocking TIF-IA degradation not only blocked nuclear translocation of NF-κB/RelA in response to aspirin and CDK4 inhibition, but also blocked the apoptotic effects of the agents [4]. The mechanism by which stress-mediated nuclear translocation of NF-κB promotes apoptosis has been the subject of debate. However, recent studies indicate nucleolar sequestration of NF-κB proteins, particularly RelA, plays an important role [5].

## 4. Nucleolar Sequestration of RelA and Apoptosis

Cellular stress not only causes a dynamic flux of regulatory proteins out of nucleoli, but also sequestration of such proteins in the organelle [65,66,67]. This sequestration regulates gene expression, impacts nuclear structure, modulates specific apoptotic pathways, and influences autophagy [68]. Examples include nucleolar accumulation of p53, LC3II and ubiquitinated proteins in response to proteasome inhibition [65,66,69,70]. Nucleolar sequestration of NF-κB repressing factor in response to heat stress, which causes repression of rDNA transcription [68], and nucleolar accumulation of von Hippel-Lindau protein, DNA methyltransferase 1 (DNMT1), and the DNA polymerase subunit POLD1 (all with a specific nucleolar detention sequence) in response to heat shock, hypoxia, and acidosis [67,71]. Most recently, Gupta et al. demonstrated regulated nucleolar compartmentalization of the histone modifier, H2B [72]. Hence, sequestration of proteins within nucleoli is also emerging as an important mechanism for maintaining cellular homeostasis.

When exploring the mechanisms by which nuclear translocation of NF-κB induces apoptosis, it was found that in response to specific pro-apoptotic stress stimuli (e.g., aspirin, serum deprivation, and UV-C radiation), the RelA component of NF-κB translocates from the cytoplasm to the nucleoplasm and then to nucleoli, causing an accumulation of the protein in the organelle [5]. Nucleoplasmic to nucleolar translocation of RelA was found to be dependent upon an N-terminal nucleolar localization signal (NoLS). Using a dominant-negative mutant deleted for this motif, it was shown that nucleolar sequestration of RelA is causally involved in reduced basal NF-κB transcriptional activity and the induction of apoptosis (Figure 3) [5]. Since this initial study, nucleolar sequestration of RelA has been observed in a number of other models. Loveridge et al. demonstrated that the NSAIDs sulindac, sulindac sulphone, and indomethacin induce nucleolar translocation of RelA in colon cancer cell lines, demonstrated that this was dependent on the N-terminal NoLS and showed that blocking nucleolar translocation of RelA blocked the apoptotic effects of these agents [63]. The anti-tumor agent, 2-methoxyestradiol (2ME2) (a naturally occurring derivative of estradiol), the potent Trk inhibitor and anti-tumor agent, K252a, [73] expression of the homeobox transcription factor, Hox-A5 [74], small molecule inhibitors of CDK4 [75,76] and the proteasome inhibition [77] have also been shown to induce nucleolar sequestration of RelA, which is associated with, or causally involved in, the induction of apoptosis. Nucleolar sequestration of p50 has also been reported. Dadsetan et al. demonstrated that the anti-TNF therapy, infliximab, induces “massive” nucleolar localisation of NF-κB/p50 in the hippocampus of rats with a portacaval shunt (PCS). They also demonstrated that this nucleolar localization is associated with a decrease in transcription of NF-κB target genes and a reduction in neuroinflammation [78]. Subsequent studies have demonstrated that nucleolar translocation of RelA, is dependent upon ubiquitination, facilitated by the multifunctional protein, COMMD1(Figure 3) [77,79].

It was originally assumed that nucleolar translocation of RelA mediates apoptosis because the protein is sequestered away from the promoters of anti-apoptotic genes. However, it is now known that once in the nucleolus, RelA triggers a cascade of events that actively promotes apoptosis (Figure 3) [6]. That is, nucleolar RelA causes nucleophosmin (NPM)/B23 to relocate to the cytoplasm, bind BAX then transport BAX to the mitochondria to initiate apoptosis [6,80,81]. Others have also demonstrated that NPM relocalization, and an NPM-BAX interaction, is critical for the pro-apoptotc effects of NF-κB stimuli such as UV-C, [81]. Interestingly, stress stimuli such as aspirin and UV-C that utilize this pathway to induce cell death, also cause degradation of TIF-IA and atypical nucleolar stress, suggesting that these initial effects on nucleoli may prime cells for subsequent nucleolar accumulation of RelA and cytoplasmic translocation of NPM (Figure 3).

## 5. Therapeutic Relevance of Crosstalk between Nucleoli and the NF-κB Pathway

High levels of nucleolar activity are a hallmark of cancer and contribute to tumor growth by allowing de-regulated protein synthesis and uncontrolled activity of nucleolar cell growth/death pathways [16,82]. Changes in nucleolar morphology and function are also common in age related neurodegenerative disorders and increasing evidence suggests that this dysfunction contributes to disease progression, as well as the normal aging process [17,18,46,83,84]. Similarly, dysregulated NF-κB activity is common in cancer, neurodegenerative disorders and aging and contributes to the progression of these diseases/aging through promotion of a chronic inflammatory environment and modulation of genes that regulate cell growth/death [40,85,86]. Hence, both these pathways are attractive therapeutic targets.

One agent that has been found to target both these pathways simultaneously through nucleolar-NF-κB signaling is aspirin [4,5]. Overwhelming evidence indicates that aspirin and related agents have considerable anti-tumor activity and the potential to prevent colorectal and other cancers. Indeed, a recent meta-analysis suggested that continual aspirin use could reduce colorectal cancer risk by up to 40% [87,88,89,90]. Epidemiological and experimental evidence also suggests aspirin use protects against neurological disorders such as Alzheimer’s disease [90,91]. However, the agent cannot be recommended for preventative purpose due to its side effect profile.

In experiments aimed at understanding the mechanism of action of aspirin against colorectal cancer, it was found that the agent causes degradation of TIF-IA and inhibition of rDNA transcription [4]. Furthermore, it was shown that this degradation is causally linked to stimulation of the NF-κB pathway, nucleolar sequestration of RelA, repression of NF-κB activity and the induction of apoptosis (Figure 3) [4,5,60]. A link between TIF-IA degradation and NF-κB signaling was observed in multiple colon cancer cells lines, in cell lines derived from human pre-malignant intestinal lesions and in four out of seven human tumors treated ex vivo with low doses of the agent, suggesting pharmacological relevance [4]. In contrast to aspirin, the small molecule PolI inhibitor CX5461, which has shown considerable promise as an anti-cancer therapy and is currently in clinical trials for hematologic malignancies and triple negative breast cancer [82,92,93], had no effect on NF-κB signaling. Similarly, the small molecule PolI inhibitor, BMH-21, that is also showing promise as an anti-cancer agent [94], did not stimulate the NF-κB pathway. These data highlight the complexity of targeting nucleoli in cancer and the differential downstream consequences. They also reveal a novel and exciting mechanism of action of aspirin that warrants further investigation.

Increased CDK4 activity is a common occurrence in cancer and contributes to cancer progression by allowing unrestricted proliferation of tumor cells [76,95,96]. In keeping with this critical role, small molecule CDK4 inhibitors (CDK4i) have shown considerable promise as anti-cancer agents and are currently in phase I/II clinical trials in a variety of malignancies. However, their precise mechanism of action is unclear. Previous studies from this lab had demonstrated that small molecule CDK4 inhibitors stimulate the NF-κB pathway and that this is essential for their pro-apoptotic activity against colorectal cancer cells [75]. More recently, Chen et al. demonstrated that CDK4 inhibition causes degradation of TIF-IA, which is causally linked to stimulation of the NF-κB pathway and the induction of apoptosis (Figure 3) [4]. These data identify small molecule CDK4 inhibitors as another class of agents that simultaneously inhibit rDNA transcription and NF-κB activity. Responses to aspirin are generally restricted to colon cancer cells [97]. In contrast, CDK4 inhibition caused TIF-IA dependent NF-κB pathway stimulation in multiple cell types, suggesting crosstalk between these pathways may be broadly relevant for the maintenance of cellular homeostasis and the induction of apoptosis.

## 6. Summary

Both nucleolar and NF-κB pathways play a vital role in maintaining cellular homeostasis under conditions of stress. Both pathways are also implicated in aging and are dysfunctional in age related diseases such as cancer and neurodegenerative disorders. Emerging evidence indicates that there are multiple levels of crosstalk between these two pathways that are important for maintaining cellular homeostasis and regulating apoptosis. However, this evolving field is in its infancy and there are still a number of important questions to be answered. For example, in what contexts is this novel nucleolar stress response pathway active and does it contribute to the aetiology of age related disease. With regard to this point, it is interesting to note that both nucleoli and NF-κB are dysfunctional in senescence, a hallmark of aging. Further understanding of the mechanisms that regulate the stability of TIF-IA, and those that link altered TIF-IA levels to activation of the cytoplasmic NF-κB pathway, would allow development of small molecules that act to specifically and simultaneously target dysfunctional rDNA transcription and NF-κB activity. Similarly, identification of nucleolar pathways triggered by RelA would allow the development of RelA mimetics that mediate apoptosis by targeting dysfunctional nucleoli. Indeed, further understanding in this area could reveal a whole new class of targets to be exploited for therapeutic purposes. It could also reveal biomarkers of response to aspirin and CDK4 inhibitors, which have already been shown to utilize nucleolar-NF-κB signaling to act against cancer cells.
